# Climatic Niche Model for Overwintering Monarch Butterflies in a Topographically Complex Region of California

**DOI:** 10.3390/insects9040167

**Published:** 2018-11-20

**Authors:** Ashley Fisher, Kiana Saniee, Charis van der Heide, Jessica Griffiths, Daniel Meade, Francis Villablanca

**Affiliations:** 1 Biological Sciences Department, Cal Poly State University, San Luis Obispo, CA 93407, USA; afishe08@gmail.com (A.F.); ksaniee@calpoly.edu (K.S.); charisvdh@gmail.com (C.v.d.H.); 2 Althouse and Meade Inc., 1602 Spring St., Paso Robles, CA 93446, USA; jessicag@althouseandmeade.com (J.G.); dan@althouseandmeade.com (D.M.)

**Keywords:** monarch butterfly, overwintering, climatic niche model, species distribution model, Western Monarch Thanksgiving Count, Maxent

## Abstract

We use climatic conditions that are associated with known monarch butterfly overwintering groves in California to build a Maxent model, and focus on the fine scale probability of overwintering grove occurrence in a topographically complex region of the state (Santa Barbara County). Grove locations are known from recent and historical surveys and a long-term citizen science database. The climatic niche model performs well, predicting that overwintering habitat is most likely to occur along the coast and at low elevations, as shown by empirical data. We then use climatic variables in conjunction with climate change scenarios to model the future location of overwintering habitat, and find a substantial shift in the predicted distribution. Under a plausible scenario, the probability of occurrence of overwintering habitat directly reflects elevation, with coastal regions having a reduced probability relative to today, and higher elevation sites increasing in probability. Under a more extreme scenario, high probability sites are only located along ridgelines and in mountaintop regions of the county. This predicted shift in distribution is likely to have management implications, as sites that currently lack monarchs may become critical to conservation in the future. Our results suggest that estimating the size of the western overwintering population in the future will be problematic, unless annual counts compensate for a shift in the distribution and a potential change in the number and location of occupied sites.

## 1. Introduction

### 1.1. Monarch Butterfly Overwintering, Population Decline, and Climate

Migratory monarch butterflies in North America are composed of two distinct overwintering populations, Eastern and Western monarchs. Most biologists and citizen scientists familiar with monarchs concede that these migratory populations are becoming less abundant [1,2,3]. Schultz et al. [3] use historical Western monarch records dating back to the 1980s to show an unambiguous decline, a negative population growth rate, abundance in the 2000s as low as 5% of the abundance in the 1980s, and an extinction probability greater than 50%. An analysis by Flockhart et al. [4] concluded that Eastern monarchs are also in decline. As a consequence, the U.S. Fish and Wildlife Service has been petitioned to list the monarch butterfly as threatened.

Monarch butterflies are a geographically wide-ranging, migratory species [5,6,7]. A wide-ranging species in decline is potentially difficult to manage, in part because there are geographically disparate, or in this case international, habitat components to understand and manage [8]. There is a need to understand one of those habitat components: the overwintering habitat of monarch butterflies. Eastern monarchs, monarchs that breed east of the Rocky Mountains, overwinter in the mountaintops of the Trans-volcanic region of central Mexico where they cluster on Oyamel fir (*Abies religiosa*) [5,9]. The Oyamel forest is comprised of thirteen high-elevation “sky islands”, with typically nine of the islands supporting up to 30 overwintering colonies [6]. The sky island distribution is thought to be climatic in origin. Today’s fir forests are relicts of boreal forests from the last glaciation, and since the glaciers retreated these forests have been displaced upward by temperate and tropical forests, and now exist in isolated islands at elevations between 2400 and 3600 m [10]. Western monarchs, monarchs that breed west of the Rocky Mountains, overwinter along the Pacific coast of California [11,12,13]. In California, overwintering is dispersed and occurs in over 400, primarily coastal, groves composed of Monterey pine (*Pinus radiata*), Monterey cypress (*Cupressus macrocarpa*), eucalyptus (*Eucalyptus* spp.), and/or coast redwood (*Sequoia sempervirens*) [1,14]. 

In spite of the distinct geographic difference between overwintering on mountaintops versus overwintering near the coastline, few authors have dwelled on this difference. One possible hypothesis for the alternative habitat use is that Eastern and Western monarchs are genetically subdivided populations with alternative climatic niche adaptations. However, Eastern and Western migratory populations have been found to represent one genetic population with admixture [7,15]. An alternative hypothesis, though no one has addressed it, is that even though migratory monarchs are not genetically subdivided, and thus share genotypic variation, they could experience phenotypic (proteomic) plasticity, leading to alternative climate niche adaptations. Both of these hypotheses would require some intrinsic differentiation, either genomic or proteomic. There are two opposite alternatives, both based on extrinsic differentiation. We suggest here that there may be differential geographical expression of a singular climatic niche. Under this hypothesis, we suggest both populations are selecting the same climatic conditions, but those conditions are found in geographically distinct settings (coastal versus mountain). Alternatively, the populations could be occupying different realized subsets of the same fundamental niche, where the two realized niches are located in distinct settings. One prediction of these extrinsic differentiation hypotheses is that climate change, in particular, warming, could lead to a modification of the coastal Californian overwintering range (making it montane or even mountain top), as is thought to have occurred in Mexico [10]. A shift from a coastal to a mountain top distribution would preclude the need for intrinsic differentiation. Instead, it would suggest the existence of a singular niche, or convergence of the realized niches. Thus, understanding the overwintering habitat of Western monarchs may allow us to better manage it, while also allowing us to explore mechanisms behind the paradoxical difference in the geography of overwintering Eastern and Western monarchs.

Multiple studies demonstrate that organismal distributions are linked to climatic conditions (e.g., [16,17,18]) and that in butterflies and moths there are direct and indirect correlates to climate, especially through host plant mediated processes [19,20,21,22,23,24,25]. The breeding ranges of both Eastern and Western monarch populations are predicted to shift northward [26] via climate change modifications to the geographic distribution of milkweed, the monarch’s obligate host. What about the geographic distribution of groves used by overwintering monarchs; will these be impacted by climate change? It is possible that the geographic range of the tree species that comprise overwintering groves in California could shift. But, it is also possible that conditions in monarch overwintering groves are direct or indirect correlates of climatic variables, such as temperature and precipitation. If so, then it is very likely that the overwintering range of monarch butterflies in California will shift. Unfortunately, it is also possible that proximity of overwintering sites to the ocean is essential, and that suitable climatic conditions will simply not be found away from the coastline. A test of this hypothesis (i.e., a climate niche model of overwintering locations as potentially modified by climate change), has yet to be constructed for monarch butterflies overwintering in Mexico or California. Responsible and climate resilient management would need to consider the geographic location of both current overwintering sites and predicted location(s) of future sites (under realistic climate change scenarios).

### 1.2. Attributes of Occupied Monarch Butterfly Overwintering Groves

Studies of habitat attributes at monarch butterfly overwintering groves [27,28,29,30,31] suggest a number of attributes, such as wind speed, light intensity, and proximity to drinking water and nectar, are important. Though these studies are useful, we find them limiting on three scales.

First, they confound conditions that are internal and external to groves. For example, it seems intuitive that wind speed at an overwintering site is determined by the combined effects of trees internal to the grove and other conditions (even trees) external to a grove; likewise, for light intensity. But, as currently interpreted, wind speed and light are not partitioned into internal and external components, but are instead attributed entirely to the internal structure. Thus, for these attributes, we do not know the relative contribution of internal (grove) and external (landscape) conditions. In contrast, water and nectar may be geographically located within a grove, outside of a grove, or both, and can thus be partitioned. Therefore, we use a species distribution model (SDM) to assess whether variables are non-randomly associated with individual overwintering grove locations. Modeling the microhabitat conditions within those groves is a separate analysis (not being conducted here), where the focal unit would not be the grove, but rather the clusters of overwintering monarchs themselves.

The second limiting scale is spatiotemporal. Currently, attributes (e.g., temperature) that have been suggested [28,30,31,32] are defined by the mean or maximum value of those attributes. They are defined as absolutes, irrespective to variation across the overwintering season. Yet, the overall mean or maximum values of an attribute might not be as important as the spatiotemporal characteristics [33]. There is even evidence supporting a temporal component to relevant habitat attributes in monarchs. Some overwintering sites are occupied during the early part of the season (October and November), and abandoned later in the season [14,30], suggesting that seasonal mean values alone are not appropriate variables. Stenseth and Mysterud [34] emphasize that it is essential to identify relevant temporal and spatial scales at which to study the effects of climate when intending to study climate change. It seems intuitive to consider spatial and temporal variability (and not seasonal means or maximums) in attributes that potentially correlate with occupied overwintering habitat. We take a spatiotemporal approach here by utilizing climatic data partitioned by month across the overwintering season. In this manner, we aim to identify relevant parameters, and simultaneously identify when they are relevant to overwintering monarch presence.

There is a balance to the logic proposed by Stenseth and Mysterud [34], given that SDMs can be over parameterized, especially when parameters are highly correlated. Such models lack generality and predictive power across temporal and spatial scales [35]. Models that describe a single (current) phenomenon well, especially when they employ a large number of parameters, are poor at extrapolating to future (unseen) data states [36]. In other words, highly parameterized models may be good at generating predictions specific to current conditions, but they fail to generate accurate probabilities of presence given future climate conditions [35,37,38]. So, although we want to identify spatial and temporal variability in potentially relevant attributes, we cannot do so if that leads to selecting highly correlated attributes [35,37]. Herein, in order to avoid over parameterization, we select sets of relevant attributes that lack correlation, and more generally balance goodness-of-fit with model complexity [35,37,38,39].

The third limitation is whether we expect monarch butterfly overwintering sites to have a static, or a dynamic (i.e., changing) distribution over time [35,36]. That is the two-step question that we ask here. Does our climatic niche model adequately represent the current distribution of monarch overwintering sites? If so, where are those climatic conditions predicted to be in the future? Previous studies [28,30,31,32] have identified a number of attributes that are associated with monarch overwintering groves. To be clear, we do not test those attributes here. Instead, we test climatic variables as predictors. Unfortunately, the GIS data layers for the variables proposed by previous works [28,30,31,32] are not available (i.e., nectar sources), or have not been projected to future dates (proximity to water, wind speed and light intensity within groves). Thus, even if the limitations pointed out above did not apply, it would still not be feasible to predict the spatial value of those attributes into the future. Therefore, we target variables suggested as relevant [28,30,31,32], but only select layers for climatic variables that are available for download (see methods), because their expected future values, projected under multiple climate forcing scenarios, are also available (see methods). Consequently, we evaluate whether climate variables are suitable for predicting where monarch butterfly overwintering groves occur now and in the future. 

### 1.3. Santa Barbara County Is a Powerful Case Study

Herein, we assess the probability that the location of conditions associated with monarch overwintering sites in California could shift in response to climatic conditions. Specifically, we test (1) whether there are non-random climatic conditions associated with overwintering sites occupied by monarch butterflies in California; (2) whether we can use the conditions to reliably predict where occupied overwintering groves occur today; (3) and, if so, whether predicted locations with these conditions in the future are different from the current locations of overwintering groves. The result of our state-wide analysis will be presented elsewhere. Here, we focus on Santa Barbara County as a powerful case study.

Sakai et al. [40] conducted a statewide survey of potential overwintering sites in California and determined sites that were occupied. Sakai and Calvert [27] conducted a similar survey on public lands. Subsequently, additional Santa Barbara County overwintering sites have been identified [41,42], reported by the general public, or identified by citizen scientists. Indeed, the number of overwintering sites in Santa Barbara County is exceptional and worthy of a case study. The coastline of California (excluding Del Norte and Humbolt Counties where Monarchs are not monitored—see methods) is 1091 km long. The coastline of Santa Barbara County is 144 km long, or 13% of the state’s coastline. Yet, of 322 overwintering sites regularly monitored for monarchs (see methods) 110, or 34%, are in Santa Barbara County. This density of monitored sites provides an unsurpassed density of empirical data against which to evaluate SDMs. Santa Barbara County also provides topographical complexity. For example, the high point is 2070 m tall Big Pine Mountain which is less than 32.2 km from the coastline. Thus, in terms of location data, occupancy data, and landscape, Santa Barbara County should be an informative case study regarding overwintering habitat. Indeed, our primary conclusions are that the climatic niche model performs well, predicting the current geographic distribution of known overwintering sites. Importantly, we find a substantial shift in the predicted distribution of overwintering sites in the future, with the probability of occurrence of overwintering sites directly reflecting elevation under a modest climate change scenario, and high probability sites being restricted to a few mountain tops in the county under a more extreme scenario.

## 2. Materials and Methods

### 2.1. Distribution Data and Models for Species That Aggregate

**Figure 1:**
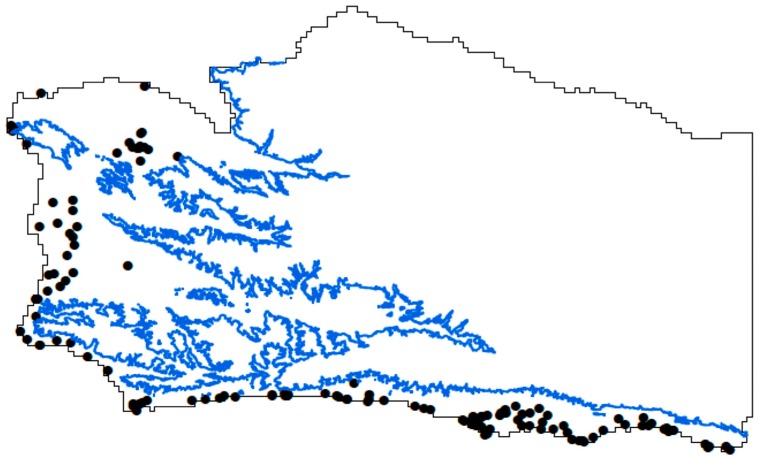
Santa Barbara County (black margin), 800-foot elevation contour (inscribed blue border), with approximate location of all known overwintering groves (solid black circles) in Santa Barbara County (excluding the Channel Islands).

We obtained georeferenced locations of monarch butterfly overwintering groves within California from the Xerces Society for Invertebrate Conservation, where locations are based on multiple surveys [27,40,41,42]. Occurrence data are largely based on the Western Monarch Thanksgiving Count (WMTC, www.westernmonarchcount.org). We included all sites whether they currently supported overwintering monarchs or not (Figure 1), as consistent with the Maxent model [43] that uses all available (current and historical) occurrence data. Given the survey effort by multiple groups or researchers [27,40,41,42], plus the sheer number of people involved and the duration of the WMTC (1997–present), we regarded the database as representative of the true geographic distribution of overwintering sites. 

Clustered (e.g., within groves) species occurrence records, those not uniformly or randomly distributed, are biased if the spatially aggregated records result in the same location being counted multiple times [44]. “Systematic sampling” sensu Fourcade et al. [45] reduces this bias. As applied here, occurrence data are reduced to individual occurrences, where the center of the overwintering grove is the single record. Therefore, our analysis is of the climatic conditions that are associated with overwintering groves (sites) and not individual overwintering monarchs or clusters of monarchs. A potential weakness of systematic sampling is that spatially aggregated records may better reflect an area’s ecological value, which thus may be underestimated [45]. Yet, alternative approaches, such as using the grove’s area or the monarch’s abundance to designate the number of records, are dangerous in practice and theory. Practically, 9.4% of the sites in the county have poorly defined perimeters, such that area estimates converted to occurrence records perpetuate error. Also, in theory, grouping multiple presence points increases spatial autocorrelation [44]. High autocorrelation of records can result in violating the assumption that residuals are identically distributed and independent, and increase the probability of a Type 1 Error [44,45]. For these reasons, we used a single point (the center) per grove.

Finally, the fact that monarch overwintering sites are currently not expected to occur above 800 ft elevation (Figure 1) [27,40,41,42] could have resulted in an apparent bias. This bias in the geographic sampling (area from which environmental data are drawn) of presence records [44] could have mimicked unequal sampling effort across the study area [45], in which case a species might be predicted to occur in only a subset of the locations where it actually occurs. However, we considered this distribution, though appearing to be geographically biased, to be representative of the true geographic distribution of overwintering groves, and did not use a correction method [45]. We used California as the background extent for our analysis. We chose this because the background extent should include regions where monarchs are equally likely to reach even if they do not currently overwinter there [46]. Biologically this makes sense because monarchs can and do range outside of the county, and away from the coast. But, to provide the fine scale analysis we seek here, we built the models for the state and then clipped the county from them, to better depict the probabilities of presence on the county scale. In this manner, our results are an example of the potential local scale (e.g., county) implications for a geographic area that is exceptionally well-studied. A larger, state-wide analysis, with its own implications, is part of a separate analysis.

### 2.2. Species Distribution Models

We built classical species distribution models [36]: they draw from all climatic variables expected to provide predictive information about the current location of overwintering groves [27,40,41,42]. The benefit is that such models should be predictive of current habitat occupancy/suitability. The cost is that the models likely cannot predict the future location of overwintering habitats because they are over parameterized, and thus non-generalizable (the specific combination of conditions may actually not occur in future locations) and are burdened with spatial autocorrelation [35,37,38]. In addition, some of the relevant variables have not been projected (i.e., there is no spatially explicit prediction of their future values available). Therefore, we constructed a second set of models using more limited sets of climatic variables, but with values that have been projected (under different climate change scenarios). These models are constrained for over parameterization, thus have the potential for generality, and are therefore useful in predicting the distribution of overwintering habitat today and likely for extrapolating to future locations [36]. The cost of generality is potentially a reduction in the ability to precisely predict the location of today’s groves given the reduced number of variables. The value of the classical SDM thus becomes apparent. 

### 2.3. Attribute Layers in Present-Day Analysis

All data for modeling the current and generalizable climatic niches are from WorldClim 2.0 [47], with resolution at 30 arc seconds (~1 km^2^) [48]. But, rather than mean annual values, we obtained monthly values across the overwintering season (defined as five month from October to February). The seven (available) variables, thought to have qualitative or quantitative association with overwintering groves [28,30,31,32], were: solar radiation (W/m^2^), water vapor pressure (kPa), total precipitation (mm), wind speed (m/s), mean, minimum, and maximum temperature (°C). Thus, models could employ up to 35 layers.

### 2.4. Attribute Layers in 2050 Analysis

We used WorldClim 1.4 data to project to the year 2050 [48]. The three (available) relevant projected variables included monthly average minimum and maximum temperature (°C) and monthly average total precipitation (mm), across the five overwintering months (October–February). Thus, predictive models could have employed up to 15 layers.

We ran the 2050 analyses with layers generated under Community Climate System, considering two emission scenarios: The Representative Concentration Pathway (RCP) 4.5 and 6.0. RCP4.5 is characterized as a “medium stabilization scenario” where radiative forcing stabilizes at 4.5 W/m^2^ (~650 ppm atmospheric CO_2_) after the year 2100 [49]. RCP6.0 radiative forcing stabilizes at a higher 6 W/m^2^ (~850 ppm atmospheric CO_2_) after 2100. The RPC6.0 pathway is less likely as it reflects a future with no established emissions policies [49].

### 2.5. Controlling Model Complexity

Maxent model predictions and model fit can be divergent depending on the model complexity allowed, which is a function of the regularization coefficient (β) [46]. Oversimplified models predict equal probabilities of presence across an overly broad range of environments [36]. Overfitted (i.e., overparameterized) models excessively constrict what qualifies as suitable habitat (due to model noise) and reduce model transferability to other (future) settings [36,46]. Optimizing β prevents overfitting by penalizing models proportionally to the number of coefficients included in the model [43]. Additionally, including highly correlated variables both increases model complexity and can lead to erratic and unpredictable projections if future variable correlations differ from the original relationships [35]. Therefore, to reduce overparameterization, optimize the regularization coefficient, and eliminate auto-correlations between layers, we followed Warren et al. [35], as outlined below.

### 2.6. Optimal Regularization Coefficient for the Base Model

Following Warren et al. [35], we ran 10 replicates with WorldClim 1.4 or 2.0, and β from 1–7, in increments of 1. We generated AICc scores with ENM Model Selection Tools, and averaged over the 10 replicates. Then, we bracketed the best β in increments of 0.1, in order to find β with the lowest AICc. 

### 2.7. Eliminating Correlated Variables

To eliminate layers with high spatial autocorrelation, we employed two methods. One followed Warren et al. [35] exactly: we first, calculated the spatial correlation of each layer to each other layer [50], second, we ran a “base model” (above) that included all layers under the optimized β, and eliminated variables with less than five percent contribution to the base model, third, we identified a “top variable” or layer with single highest percent contribution, and eliminated any variables with a correlation coefficient 0.7 or higher with the “top variable”, fourth, we ran the model again, minus eliminated variable, and top variable from that cycle, and repeated the elimination process until every variable layer was eliminated, or added to the “top variable” list. These models were exclusively composed of “top variables” minus their correlated variables. Our second method was a relaxation of Warren et al. [35]. It avoided potential over-elimination of layers, which could result in under-parameterization and an overly generous estimate of the probability of presence [36]. We did not eliminate variables with less than five percent contribute. Instead, we kept them if their correlation coefficient with the top variable was less than 0.7. Thus, variables were eliminated based on correlation, but not percent contribution. Both of these methods, referred to as the “top variable uncorrelated” and “uncorrelated” methods, were applied to the classical SDM and the 2050 models.

### 2.8. Final Models

To determine the optimal β for our final models, we followed the aforementioned layer selection processes, generated and compared the average AICc values for β values ranging from 0.1 to 2.0 (increments of 0.1), and selected the β with the lowest mean AICc. All final models were generated using the logistic output.

## 3. Results

### 3.1. Species Distribution Model—Climate Variables and Current Distribution

**Figure 2:**
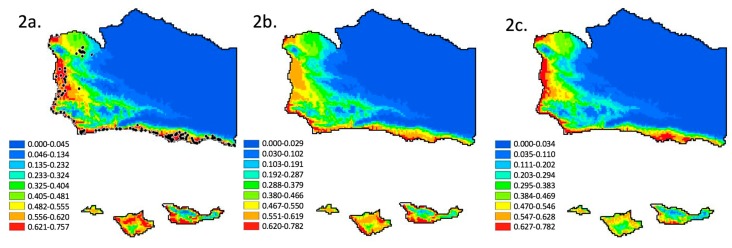
Results of MaxEnt climate niche analysis for the current distribution of monarch butterfly overwintering habitat in Santa Barbara County, California: (**a**) using an optimized regularization parameter, up to 35 WorldClim 2.0 layers, reduced by the “top variable uncorrelated” elimination method of Warren et al. [35] for reduction of over parameterization, and showing the location of known overwintering sites (as in Figure 1), (**b**) using an optimized regularization parameter, up to 35 WorldClim 2.0 layers, and the “uncorrelated” method modified from Warren et al. [35] for reduction of over parameterization, and (**c**) 35 WorldClim 2.0 layers, and no variable elimination or optimization method to reduce over parameterization, thus potentially allowing for over parameterization. Relative probabilities are indicated by the color legend. All models show similar probabilities, suggesting a current distribution in the coastal zone and extending into coastal valleys to a lesser or greater degree. Addressing over parameterization does not impact our ability to predict the current range.

The distribution of monarch butterfly overwintering sites was modeled using three approaches. Figure 2a shows a model using 35 WorldClim 2.0 layers and the method of Warren et al. [35] to exclude high autocorrelation and low percent importance variables. This “top variable uncorrelated” approach used a final β of 0.5, and the following variables: February water vapor pressure, January minimum temperature, and November mean wind speed. Figure 2b shows a model using 35 WorldClim 2.0 layers and our modification of Warren et al. [35] to exclude high autocorrelation variables. The “uncorrelated” approach used a final β of 0.3 and the following variables: February water vapor pressure, January minimum temperature, October mean wind speed, February precipitation, and October solar radiation. Figure 2c shows the results of a model using 35 WorldClim 2.0 layers and no variable elimination method or beta optimization to reduce over parameterization, thus potentially allowing for over parameterization. A final beta of 1.0 (the default) and the following variables were used for the model in Figure 2c: monthly averages for solar radiation, water vapor pressure, total precipitation, and wind speed, plus monthly mean, minimum, and maximum temperature (°C), over the five-month overwintering season.

The three models of current monarch butterfly overwintering habitat distribution effectively yield the same predicted distribution (Figure 2a–c) suggesting reduced variable models (eliminating auto correlation and low percent importance) are as effective as full variable models. The essential differences between the models are in the relative probability of occurrence assigned to any specific locality. An analysis using the exclusion criteria of Warren et al. [35] yields a predicted distribution along the coastal plains and coastal valleys of Santa Barbara County (Figure 2a). A similar distribution is predicted if the exclusion criteria are relaxed (Figure 2b). Though, in this case, the sites with the highest probability are immediately adjacent to the coast and they do not extend as far into the coastal valleys. A similar distribution, but with intermediate probabilities relative to the two previous models, results if no variables are excluded (Figure 2c). All models suggest that the current distribution is in the coastal zone and extends into coastal valleys to a lesser or greater degree (Figure 2a–c). This result is entirely consistent with the distribution documented by previous research [27,40,41,42]. In addition, all known sites (Figure 1) occur in areas with a predicted probability of occurrence of suitable climatic conditions from a low of 0.40, 0.20, and 0.30 (Figure 2a–c, respectively) to a high of 0.75, 0.78, and 0.75 (Figure 2a–c, respectively). Probabilities of presence were generated using the full range of conditions and grove presences within California. Therefore, the focus here should be on the relative difference in probability between locations, rather than the absolute value of the probabilities. Also, models that exclude variables (Figure 2a,b), select water vapor pressure in February and minimum temperature in January in both cases. They both select average wind speed, but in different months (October versus November).

### 3.2. Species Distribution Model—Moderate Climate Change Scenario

**Figure 3:**
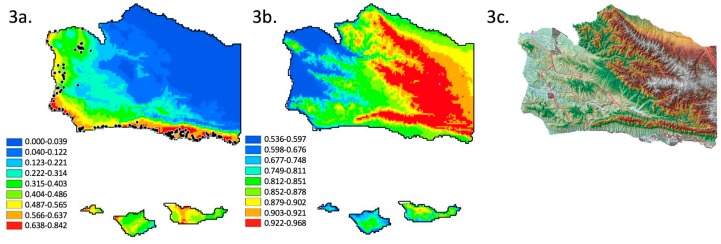
Results of a MaxEnt climate niche model analysis for the current and future distribution of monarch butterfly overwintering habitat in Santa Barbara County, California, generated using an optimized regularization parameter, and two elimination methods for reduction of over parameterization which converge on the selected layers: (**a**) analysis for the current distribution using, WorldClim 2.0 layers, and showing the location of known overwintering sites (as in Figure 1) and, (**b**) corresponding 2050 extrapolated distribution, calibrated using WorldClim 1.4 layers under the RCP4.5 projection, and (**c**) relief map of the county. Relative probabilities for each model are indicated by separate color legends, with (**b**) showing overall higher relative probabilities. The difference between present and extrapolated maps generated by this model suggests the current coastal zone/coastal valleys distribution will be replaced by a higher elevation distribution, where the probability of suitable climatic conditions will be proportional to elevation.

The current distribution of monarch butterfly overwintering locations (Figure 3a), using WorldClim 2.0 layers, two layer selection methods (that converge on minimum temperature in January and precipitation in February), and the RCP4.5 base, is essentially the same coastal distribution seen in Figure 2. High probability sites are adjacent to the coastline and lower probability sites are in coastal valleys (Figure 3a). This result is less specific (or more general and “blurred”) relative to Figure 2, as expected given a reduction to two variables [36]. The resultant distribution is different under the modest [49] RCP4.5 climate change scenario, calibrated by WorldClim 1.4 data, β of 0.2, minimum temperature in January, and precipitation in February (both layer elimination methods converge). In contrast to Figure 2 and Figure 3a, the 2050 projection shifts the probability of occurrence geographically (Figure 3b). The probability of occurrence of overwintering habitat (Figure 3b) becomes essentially proportional to elevation (Figure 3c), with the lowest probability of occurrence of overwintering habitat (0.53) along the coastline and associated coastal valleys, and the highest probability of occurrence (0.97) over interior mountains (Figure 3c). Indeed, the relief map of Santa Barbara County elevation (Figure 3c) is almost identical to the overwintering habitat probability map (Figure 3b). In addition to the geographic shift, the total area in Santa Barbara County with a high probability of presence is projected to increase substantially, according to this model’s 2050 projection (Figure 3).

### 3.3. Species Distribution Model—Extreme Climate Change Scenario

**Figure 4:**
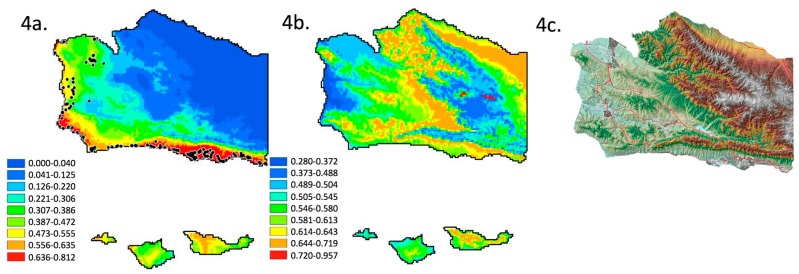
Results of a MaxEnt climate niche analysis for the current and future distribution of monarch butterfly overwintering habitat in Santa Barbara County, California: (**a**) model for the current distribution using an optimized regularization parameter, WorldClim 2.0 layers, and two elimination methods for reduction of over parameterization which converge on the selected layers, and showing the location of known overwintering sites (as in Figure 1) (**b**) model for the future distribution using an optimized regularization parameter, WorldClim 1.4 layers under the RCP6.0 projection, and two elimination methods for reduction of over parameterization which converge on the selected layers, and (**c**) relief map of the county. The difference between present and extrapolated maps generated by this model suggests the current coastal zone/coastal valleys distribution will be replaced by a more complex distribution where the probability of suitable climatic conditions relate to, but are not proportional to elevation. Instead, it is bimodal (low near the coast, higher in coastal ranges, low again on the slopes of interior ranges, and highest on mountain tops). A cluster of mountain top locations would be the highest probability sites in the entire county (middle of right half of (**b**)).

The current distribution model of monarch butterfly overwintering locations (Figure 4a), using WorldClim 2.0 layers, projected from the RCP6.0 base model, beta of 0.2, and January minimum temperature, and February precipitation (both exclusion methods converge), is essentially the coastal distribution in Figure 2a–c and Figure 3a. High probability sites are adjacent to the coastline and to a lesser degree in coastal valleys (Figure 4a). This result is less specific than Figure 2, given a reduction to two variables [36]. In contrast to Figure 4a, the probability of occurrence of suitable overwintering conditions in the 2050 projection of the RCP6.0 scenario (Figure 4b) substantially shifts the probability of occurrence geographically. The lowest probability of occurrence of the overwintering habitat is along the Western coastline, associated coastal valleys, and some interior mid elevation slopes in Santa Barbara County (Figure 4b). The positive correlation between elevation and probability of occurrence seen in Figure 3b,c is not present. Instead, higher probability sites are along the ridgeline of coastal ranges (first range North of the ocean Figure 4b,c), lower on intervening valleys (Figure 4b,c) and mid-elevation slopes (Figure 4b,c), and highest at the very highest elevations (Figure 4b,c) in the county. This model is essentially bimodal, with highest probability sites being isolated in the center, surrounded by low probability sites, and surrounded by higher probability sites creating a ring. In addition to the geographic shift, the total area in Santa Barbara County with a high probability of presence increases in this 2050 projection (i.e., more area in Figure 4b at higher probability than in Figure 4a), though highest probabilities are concentrated at mountain top sites (Figure 4b). The RPC6.0 pathway is less likely, reflecting a future without any emissions policies [49]. Thus, only in theory, or sad state of global affairs, would we predict such an extreme geographic shift.

### 3.4. Optimizing Beta, Removing Auto Correlated and Low Importance Variables

Models aimed at estimating the future probability of occurrence of suitable climatic conditions run without optimizing the regularization coefficient, without removing correlated variables, and retaining variables irrespective of percent contribution, all yield the same result. They extrapolate a future countywide probability of overwintering habitat that is low or close to zero (results not shown—the figure is all blue). This result can be interpreted to mean the exact combination of all 35 conditions that are currently associated with overwintering groves is very unlikely to occur in Santa Barbara Co. in 2050 [36]. In contrast, Figure 2, Figure 3 and Figure 4 support optimizing fit and reducing over parameterization to provide generalizable solutions that project to a novel climate.

## 4. Discussion

In spite of using alternative subsets of climatic data, and alternative methods to address over and under parameterization, we find our climatic niche models yield robust predictions regarding the current geographic distribution of monarch butterfly overwintering habitat in Santa Barbara County. All models of the current distribution of overwintering climatic conditions show the highest probability along the coastline of Santa Barbara County, with variable probabilities in coastal valleys depending on the individual model. No model suggests that suitable overwintering conditions currently exist in the mountainous interior of the county. Therefore, given model fit to the abundance of known monitoring data, we assume climate niche models can extrapolate the future location of suitable overwintering conditions. In contrast to the current distribution, the projection of climate variables using modest [49] climate forcing RCP4.5 geographically shifts overwintering conditions inland and away from the coast. Under this climate forcing scenario, the probability of occurrence of suitable overwintering conditions becomes roughly proportional to elevation. Using a more extreme climate forcing RCP6.0 [49] projection, there is a higher probability that overwintering conditions are located on lower elevation coastal slopes and mountaintops, while intermediate (and intervening) elevations and the coastline and coastal valleys, all have lower probabilities. Therefore, our models suggest that climate change will result in an inland and upslope displacement of suitable overwintering conditions. If climate follows a trajectory that is similar to the RCP 6.0 pathway, our model suggests Santa Barbara Counties’s dispersed overwintering populations will become centralized, as seen today in the highlands of Mexico.

Fortunately, the RCP6.0 scenario is regarded as less plausible than the RCP4.5 scenario [49]. The RCP6.0 model of climatic forcing stabilizes at a higher W/m^2^ than the RCP4.5 model, reflecting less control over greenhouse gas emissions. But, whether this scenario is more or less likely should not be confused with the predicted outcome if this level of forcing were to occur. We provide evidence that the quintessential coastal distribution of overwintering sites (Figure 1, Figure 2a–c, Figure 3a and Figure 4a) could, at least in theory, convert to the quintessential mountaintop Mexican distribution, given sufficient change to climate (Figure 4b). Meaning that, even if those more extreme climatic shifts are unlikely, our analysis provides evidence that the differential distribution (coastline in California, and mountain tops in Mexico) can be explained primarily by climate. Thus, the difference in overwintering geography could be driven entirely by extrinsic factors. We hypothesize this to be the case, especially in the absence of intrinsic (e.g.,: genetic, genomic, or proteomic) evidence to the contrary (e.g.,: [7,15].)

The correlation of elevation and latitude and longitude, with climatic conditions, is the basis for the interpolated WorldClim database [47], and its use of the adiabatic lapse rate [51]. In other words, elevation and latitude are inextricably tied to climate. In the contemporary distribution, suitable climatic conditions are constrained to the coast (Figure 1, Figure 2a–c, Figure 3a and Figure 4a). In the future, under the RCP4.5 scenario, we infer that suitable climatic conditions would occur at higher elevations (Figure 3b). Thus, if monarchs currently overwinter along the coast to take advantage of cool temperatures [28,29], and if temperatures in California are predicted to rise through the year 2100 [52,53], then similarly cool temperatures, and overwintering monarchs, should be found at higher elevations later this century. Indeed, elevation may become an apt predictor of suitable climatic conditions for overwintering monarchs. If elevation in Santa Barbara County is represented as a heat map (higher elevations as warmer), then the elevation map (figure not shown) and the overwintering climatic condition probability map for the RCP4.5 model (Figure 3b) are almost identical. It is intriguing that the future distribution is predicted to be positively associated with elevation, since we do not currently see that association. If anything, we see the opposite (negative) correlation (Figure 1, Figure 2a–c, Figure 3a and Figure 4a). Thus, elevation as a useful predictor of suitable climatic conditions for overwintering monarchs in Santa Barbara County would not be a paradigm shift, it would be a paradigm inversion. In contrast, the complex and radically different distribution of high probability sites under the RCP6.0 scenario (Figure 4b) would be a paradigm shift. Regardless, both scenario outcomes (Figure 3b and Figure 4b) suggest that our focus should no longer be restricted to the coast as effects of climate change escalate.

Across the globe, distribution and abundance data are essential for directing and prioritizing conservation efforts [54]. Indeed, the petition to federally list the monarch butterfly was based upon long-term abundance and distribution data indicating that monarch populations are in decline [1,2,3,4,55]. Using data from the Western Monarch Thanksgiving Count (WMTC), coordinated by the Xerces Society for Invertebrate Conservation (www.westernmonarchcount.org), and collected from 1997–2017, Schultz et al. [3] show an unambiguous decline in the size of the western monarch overwintering population. Long-term data sets such as the WMTC and the North American Butterfly Association’s Butterfly Counts are valuable because they control for site (locations are periodically resampled), facilitating robust analyses of population trends [3].

Given the importance of long-term datasets like the WMTC, we are gravely concerned about our future ability to analyze the population trends of overwintering Western monarchs. Our concerns are two-fold. First, if coastal sites are gradually abandoned (due to lowering probability of suitable climatic conditions) then the population counts at those sites are predicted to decline. This would reduce the power of long-term analyses controlled for sample sites. For example, part of the analysis performed by Schultz et al. [3] uses site-specific counts and estimates site-specific parameters, which is possible when sites are repeatedly sampled (controlled). They state “We also assumed that each site at which monitoring took place in a given year was an independent index of the total abundance in that year”. If, in the future, site-specific abundance data reflect the combined effect of fluctuations in total abundance and gradual site-specific abandonment, then analyses of site-specific counts will not be indicative of any single population process. Unfortunately, site-specific decline is predicted to occur via gradual or sudden site abandonment by monarchs avoiding unfavorable climatic conditions, and is likely to occur in Santa Barbara County. Specifically, the proportion of the county area that is considered to be suitable in our models increases, while the absolute amount of suitable habitat in the coastal belt decreases. This geographic shift will make monitoring the Western Monarch population problematic. However, by continuing the WMTC, we may gain a better understanding of range shifts, as is occurring with Breeding Bird Survey data [56]. Furthermore, it’s pertinent to note the “current” models generated by analysis of WorldClim 2.0, have base year data from 1970–2000, meaning that this geographic shift may already be underway.

If, as suggested by Figure 3b and Figure 4b, our focus needs to shift away from the coast, then where should we focus management, conservation and monitoring? This brings us to our second concern. Though serendipity allows for the discovery of new overwintering sites that are potentially added to the WMTC, currently there is no established protocol, within the WMTC or otherwise, for the search and discovery of new (previously unknown or unoccupied) sites. Though our model can help to identify very general swaths of the county where monarchs are more likely to overwinter, further knowledge is needed in order to inform our search for new occupied overwintering sites. We do not propose to develop a protocol here. Instead, we consider several potentially relevant sources or types of knowledge.

It may be important to understand movement. Monarchs are highly mobile, migrating thousands of kilometers. Yet, to the degree that they follow migratory paths, they would only encounter and select a subset of geographic locations with suitable overwintering conditions. Dingle et al. [57] conclude Western monarchs “often follow riparian corridors” as migratory paths. Pyle [58] comes to a similar conclusion, but adding that riparian corridors are not followed if their direction is contrary to the overall direction of migration. Both imply that a subset of suitable sites might be encountered due to constrained use of migratory paths. Wenner and Harris [59] argue that a portion of Santa Barbara County’s population is not migratory, instead expanding away from the coast in summer and contracting back in winter. This suggests that migration is less constrained because it is more localized. Unfortunately, local-scale migration route maps (with routes from overwintering groves to breeding habitat) do not exist for Western monarchs. Without the benefit of any additional information, an a priori migration route map would be the North-South and East-West oriented riparian corridors. We suggest that since displacement of overwintering sites (away from the coast) is hypothesized, this hypothesis should be tested by testing the prediction that new overwintering sites would most likely be discovered in areas with a high probability of overwintering climatic conditions (e.g.,: Figure 3b) and along riparian corridors. This might inform the search for the true species distribution beyond the resolution our climatic niche models provide.

It may be important to understand habitat characteristics in order to help us recognize suitable habitat at novel sites. Sakai and Calvert [27] provide an a priori hypotheses for recognizing new sites (a) “A typical monarch overwintering site is located within a few kilometers of the ocean” (b) “The roosting site is a relatively dense grove of trees in a drainage”. (c) “The grove of trees has a well-developed understory” (d) “The grove usually has a southern exposure” and (e) “The grove of trees must be large enough so that the butterflies can adjust the position of their clusters within the grove depending upon wind patterns, sun direction, and season”. These hypotheses, though never tested directly, have been expanded upon. For example, Leong et al. [32] find “winter sites had lower light intensities, solar radiation, wind velocities, and higher vapor pressure deficit than a non overwintering grove”. Likewise, Weiss et al. [29] conducted a quantitative analysis and extrapolated a qualitative conclusion: “sites must allow sufficient insolation to allow butterflies to thermoregulate by basking in sunlight”, while “groves must also be dense enough to moderate temperature, humidity, and wind”. Unfortunately, these three groups of authors (Sakai and Calvert vis a vis Leong et al. vis a vis Weiss et al.) describe similar conditions but use different attributes in order to do so. The overlapping descriptors, with some license to interpret taken, is Sakai and Calvert’s “relatively dense grove of trees…well developed understory…southern exposure”. This seems to descriptively match the mechanistic conclusion (quoted above) reached by Leong et al., and Weiss et al.’s “forest groves must also be dense enough…sites must allow sufficient insolation”. Meaning that groves must be dense, but have gaps or opening for sufficient light.

How might we use this information to recognize new sites? The combined suite of conditions proposed by the three groups of authors could be the a priori model by which we recognize new overwintering sites. This requires that we assume the correlation among combined suite of conditions will be constant over time. Unfortunately, this is a questionable assumption given at least one of the a priori conditions (“A typical monarch overwintering site is located within a few kilometers of the ocean”) is unsupported by our models. Models of the probability of suitable climatic conditions (Figure 3b and Figure 4b) are independent of variable relationships that may only be relevant in the current climate [36]. Thus, factors that were synthesized from Sakai and Calvert [27], Leong et al. [32], and Weiss et al. [29] should definitely be considered, but they should be regarded as potentially becoming disarticulated from each other as the location of suitable climatic conditions shift. Combining these factors with probabilistic models (Figure 3b and Figure 4b) may be the most fruitful.

If nothing else, it will be important to test the prediction that some current overwintering sites are being abandoned and new sites are being established. Testing this prediction will allow for an assessment of how well analysis of the WMTC data might reflect population trends in the future. While, intelligently searching for new sites will both allow us to assess the relative value of the WMTC data, and initiate the very data collection (site discovery) that would be required to inform future Western monarch monitoring.

Groves of trees that could someday provide suitable overwintering habitat likely exist today in the upland areas of Santa Barbara County. Forty-four percent of Santa Barbara County’s land area (3136 km^2^) is comprised of the Los Padres National Forest. The Forest is located almost entirely in the Northeastern corner of the County. This corner also contains most of the County’s high elevation habitat (Figure 3c) and therefore most of the future high probability habitat (Figure 3b and Figure 4b). Currently, hardwoods and mixed conifer stands exist throughout the Forest. The long-term drought affecting California has resulted in oak mortality at lower elevations, and conifer mortality at higher elevations. In addition, the Forest contain large tracks of chaparral, which is a fire prone community. Thus, predicting the future location of hardwoods and conifers that could provide suitable overwintering groves might require tree-species specific distribution models that incorporate the effects of climate change, and will certainly require an overlays of fire history.

Like all species distribution models, ours should be interpreted with some caution. It is important to remember our models do not model the probability of occurrence of individuals or clusters. Instead, they model the climatic characteristics of sites that contain overwintering groves, in a geographic space. In other words, they answer the question, “what are the climatic characteristics of sites that contain overwintering groves”. Our approach is also a correlative species distribution model and is not mechanistic [36], meaning that we do not test physiological tolerances and then map their boundaries geographically. Instead, we assess the correlation between climatic variables and their temporal variation, relative to the geographic distribution of known groves. Thus, the determinant of whether a site is suitable for overwintering (or not) may be a climatic variable incorporated into our model, or it may be any myriad attributes that correlate with that climatic variable. The maps of the predicted future distribution should be regarded as extrapolations because they predict the range of overwintering monarchs into a space, and novel environment that has never been sampled [46]. In addition, monarchs use trees in groves for overwintering. This biotic interaction, and others, have not been modeled, and thus errors of commission (placing a species where it will not be present because the biotic interaction will not be present) are possible [60]. Though this is a weakness, extrapolated models still have value and they are essential predictors [36]. Our results provide statistically-based hypotheses to give direction for future SDM modeling and field-based research on abundance and distribution of overwintering Western monarchs. Those future snapshots of data will, in turn, test our predictions. Importantly, these same snapshots will inform how to interpret or modify the WMTC. Finally, as we have tried to outline throughout, Santa Barbara County is a fine-scale case study. We present it here given it is a powerful example of the potential impacts of climate change on the distribution of monarch butterfly overwintering locations. We also point out that, since these results are derived from a larger analysis of the distribution of overwintering sites in California, the heat maps (Figure 2, Figure 3 and Figure 4) that we present do not reflect the probability of occurrence of suitable overwintering habitat relative to Santa Barbara County, but instead they reflect the probability of occurrence of suitable overwintering habitat relative to the state. Though we focus on Santa Barbara County as the case study, due to the monarch’s high mobility and large range which spans almost the entire west coast of California, we evaluate the suitability of environmental conditions relative to the span of conditions actually available in the State. That broader scale analysis will be presented elsewhere as the results and implications are consistent with those presented here, but broader reaching.

## 5. Conclusions

Herein, we predict a significant change in the distribution of monarch butterfly overwintering habitat in Santa Barbara County based on a correlative climatic niche model, multiple greenhouse gas emission scenarios, projected climatic conditions, and multiple variable weighting and elimination protocols. The well-known coastal distribution of monarch butterfly overwintering sites in Santa Barbara County is not likely to persist. Instead, monarch overwintering habitat in Santa Barbara County, under a modest emissions scenario, is predicted to enter a phase where the probability of occurrence is a function of elevation. Under a more extreme (and less likely) emission scenario, overwintering monarchs could ultimately occupy only the highest elevation sites in the County. This extreme scenario also shows the potential, at least in theory, that climate alone could convert the distributed overwintering range seen in Santa Barbara County to a more centralized, highland range, as currently seen in the Mexican overwintering population. Finally, if monarchs modify the distribution of their overwintering sites, then compensatory modifications will need to be made to the analysis of WMTC data for assessing the size and trend of the Western Monarch population. Likewise, management at the county level will need to shift from only preserving active groves, to also searching for, managing, and conserving future ones.
